# Infrapopliteal angioplasties for limb salvage in diabetic patients: Does the clinical outcome justify its use?

**DOI:** 10.4103/0971-3026.40302

**Published:** 2008-05

**Authors:** L Dayananda, Srikanth Moorthy, KP Sreekumar, Nirmal Kumar Prabhu, TN Chetan

**Affiliations:** Department of Radiology, Amrita Institute of Medical Sciences, Amrita Lane, Elamakkara P.O, Kochi - 682 026, India

**Keywords:** Infrapopliteal angioplasty, limb salvage

## Abstract

**Purpose:**

To analyze the results of infrapopliteal percutaneous transluminal angioplasty (PTA) as a primary treatment modality in diabetic patients with non- healing ulcers or gangrene.

**Materials and Methods:**

We retrospectively studied 35 angioplasties, performed as the first line of treatment to salvage diabetic feet. The patients were followed up for 12 months.

**Results:**

Grade 1 and 2 ulcers were seen in only 20% of patients (5.71 and 14.28%, respectively); 37.14% of patients had grade 3 ulcer, and 42.8% were in the gangrene stage (grade 4 - 34.2% and grade 5 - 8.5%). A total of 77 lesions in 46 arteries (including six popliteal and suprapopliteal lesions) were dilated in 35 limbs. Of the 71 infrapopliteal lesions, 86% of lesions were classified as group C or D (group A - 9.3%, group B - 4.65%, group C - 37.2%, and group D - 48.83%). Overall technical success rate was 84.7%. The vascular complication rate was 26% (12 arteries out of 46). Clinical success was achieved in 29.1% of cases at the end of 6 months and 58.6% at the end of one year. Limb salvage rates were 79.4% at the end of 6 months and 75.8% at the end of one year.

**Conclusions:**

A high technical success rate can be achieved even in situations traditionally considered unfavorable for angioplasty. Infrapopliteal angioplasty can produce limb salvage rates comparable to bypass surgery in diabetic patients with extensive disease.

In diabetic subjects with ischemic foot ulcers, successful revascularization reduces the rate of major amputation.[1] Some consider amputation better than vascular reconstruction as it is a cheaper and quicker option. However, every attempt should be made to obtain functional limb salvage in view of the following facts:[2]

- a. Perioperative mortality for below-knee amputation is about 8-10% and that of above-knee amputation is 15-20%.
- b. Forty percent of patients die within 2 years of a major amputation. A second amputation is required in 30% of patients. Major amputations need bigger prostheses. Using bigger prostheses demands higher cardiac output from an already strained heart.
- c. Mid-term and long-term costs of amputation are higher than that of vascular reconstruction. Moreover, amputation leads to a poor quality of life.
- d. Limb salvage patients have a limited life expectancy due to associated cardiac and cerebrovascular diseases. The best endpoint for outcome might be retention of a salvaged and usable limb at death, rather than the vascular patency rate.[3]

While the effectiveness of peripheral bypass is established, that of infrapopliteal angioplasty remains debated.[4] This is because a wide range of technical success and patency rates are reported in literature. It has been observed that these poor patency rates do not correlate with the much better limb salvage rates achieved with angioplasties. This is because a limb with an ulcer has more oxygen demand as compared to a limb without tissue loss. Once the ulcer has healed, maintenance of skin integrity can be achieved with less oxygen.[5] The purpose of our study was to analyze the results of the infrapopliteal angioplasties done at our center in terms of clinical success, when performed as the first line of treatment for the salvage of diabetic feet.

## Materials and Methods

We retrospectively studied 35 angioplasty procedures performed between January 2003 and November 2006. All the patients were diabetic with non-healing foot ulcer or gangrene. Comorbid conditions of the study population are listed in Table 1. These patients underwent angioplasty as the primary modality of management. Bypass surgery was considered in these cases only when angioplasty failed or was not technically feasible. All foot ulcers were clinically graded using the Wagner's classification:[2] grade 0 - pre-ulcerative lesion, healed ulcer, presence of bony deformity; grade 1 - superficial ulcer without subcutaneous tissue involvement; grade 2 - penetration through the subcutaneous tissue (with exposure of bone, tendon, ligament, or joint capsule); grade 3 - osteitis, abscess, or osteomyelitis. grade 4 - gangrene of the forefoot; and grade 5 - gangrene of the entire foot.

**Table 1:** Comorbid conditions of study population

Condition	Number of patients
Hypertension	22 (62)
Peripheral neuropathy	25 (71)
Diabetic nephropathy	23 (65)
Diabetic retinopathy	20 (57)
Coronary artery disease	22 (62)

Figures in parentheses are in percentage

All patients underwent a diagnostic angiogram followed by angioplasty on another day. Infrapopliteal lesions on angiography were classified according to the Transatlantic Intersociety Consensus (TASC) document on the management of peripheral arterial diseases:[5] group A - single stenosis shorter than 1 cm; group B - multiple focal (<1 cm) stenoses of the tibial or peroneal arteries (including up to two focal stenoses at the tibial trifurcation) and short tibial or peroneal stenoses in conjunction with femoropopliteal disease; group C - longer stenoses of 1-4 cm and occlusions 1-2 cm in length, as well as extensive stenosis at the tibial trifurcation. Traditionally, these are most commonly treated surgically; group D - occlusions longer than 2 cm and diffusely diseased tibial vessels, traditionally considered too extensive for endovascular treatment.

All procedures were carried out the in the radiology suite under local anesthesia. Our premedication protocol includes hydration and sedation (intramuscular pethidine 50 mg and phenergan 25 mg). An ipsilateral common femoral artery antegrade puncture was performed using a micropuncture set. We used a 6F short sheath for arterial access and 4000 IU of heparin was administered through the sheath at the beginning of the procedure. The lesions were crossed using a 0.035-inch hydrophilic wire-straight catheter combination. An additional 2000 IU of heparin was administered at this point. The 0.035-inch guide wire was then exchanged for a 0.014/0.018-inch extra-stiff guide wire. We used an over-the-wire Symmetry balloon (Boston Scientific, Galway, Ireland; 2.5 mm × 40 mm or 2.5 mm × 100 mm) or monorail coronary balloon (Johnson and Johnson, Roden, The Netherland; 2.5-4 mm average diameter) for dilatation. The balloons were inflated for about 30 s during each step of the dilatation. Following deflation of the balloon, the catheter was withdrawn to the mid-superficial femoral artery. A check angiogram was performed with a wire across the lesion for access. Redilatation was done whenever required.

**Figure 1 (A, B):**
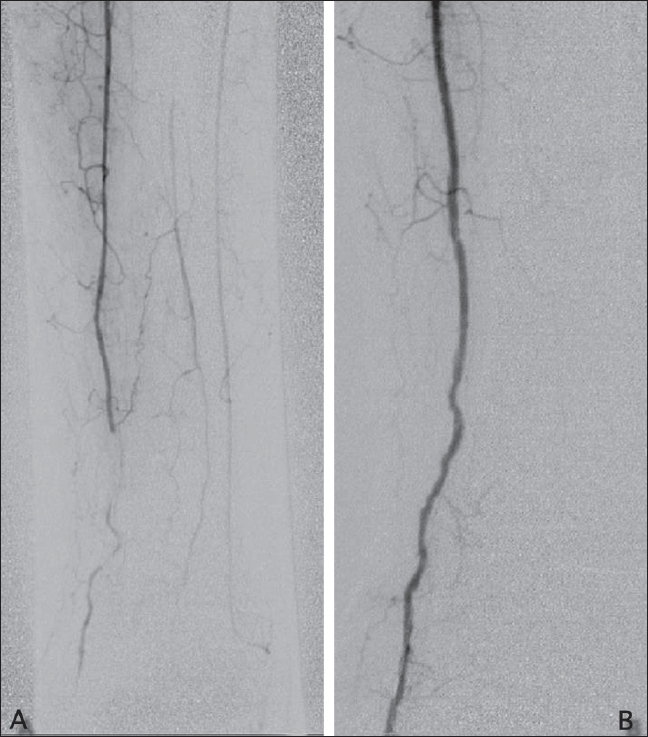
Pre- (A) and post-angioplasty (B) images of the distal anterior tibial artery showing a long-segment lesion in A, which was successfully angioplastied (B) without residual stenosis

Technical success was defined as dilatation resulting in less than 50% residual stenosis as compared to the previous angiogram [Figure 1]. 100-200 μg of intraarterial nitroglycerin was used whenever spasm was encountered. Post-angioplasty, 4000 IU of heparin was given subcutaneously, 8 hourly for 2 days following the angioplasty.

The patients were followed up at 2 week intervals by the podiatric surgeons. The patients received foot care consisting of wound dressing, minor debridments, limited amputations, infection control, and appropriate footwear. Clinical follow-up consisted of pulse examination and evaluation of the ulcer or amputation site healing or resolution of infection. Clinical success was defined as healing of ulcer or minor amputation. Limb salvage was achieved when the plantar stand (to maintain an upright position on the feet) was maintained, even if tarsometatarsal amputation had to be done.[6] Any above-the-ankle amputation was considered a failure of the revascularization procedure.

## Results

Of the 35 patients, 30 were male (85.7%), with a mean age of 64.9 years. Grade 1 and 2 ulcers accounted for 20% of the patients (5.71% and 14.28%, respectively), while 37.14% of patients had grade 3 ulcer and 42.8% had gangrene (grade 4 - 34.2%, grade 5 - 8.5%). A total of 77 lesions in 46 arteries (including 6 popliteal and suprapopliteal lesions) were dilated in 35 limbs [Table 2].

**Table 2:** Pre-procedural anatomical distribution of lesions

	ATA	PTA	Peroneal	TP Trunk	Popliteal	SFA	Total
Number of arteries involved	19	13	4	7	1	2	46
Number of lesions	37	22	4	8	1	5	77
Group A	3	-	-	1	-	-	4 (9.3%)
Group B	1	-	-	1	-	-	2 (4.6%)
Group C	7	5	1	3	-	-	16 (37.20%)
Group D	8	8	3	2	-	-	21 (48.83%)

Of the 71 infrapopliteal lesions, 86% of lesions were classified as group C or D (group A - 9.3%, group B - 4.65%, group C - 37.2%, and group D - 48.83%). Two infrapopliteal vessels were present in 51.4% of limbs (18/35) and a single vessel in 37.1% (13/35). Only 11.4% of limbs had all three infrapopliteal vessels (4/35). Most of the lesions were in the anterior tibial and posterior tibial arterial territories (44.1% and 30%, respectively). Overall technical success rate was 84.7% [Table 3]. The vascular complication rate was 26% (12 arteries out of 46) and all of them were minor. Clinical success was achieved in 29.1% of patients at the end of 6 months and 58.6% at the end of one year [Table 4]. Limb salvage rates were 79.4% at the end of 6 months and 75.8% at the end of one year.

**Table 3:** Technical success in various groups of lesions

	Technical success	Technical failure
Group A	4	0
Group B	2	0
Group C	13	3
Group D	17	4
Suprapopliteal	3	0
Total	39 (84.78%)	7(15.21%)

**Table 4:** Results of infrapopliteal angioplasty in terms of clinical success and limb salvage

Total number		Presentation		Clinical success		Limb salvage	
			
Male	Female	Ulcer	Gangrene	6 month	1 year*	6 month	1 year*
30	5	21	14	10	17	27	22
35		60%	40%	29.1%	58.6%	79.4%	75.8%

(Only 29 cases completed 1 year at end of study. One case died during follow-up)

## Discussion

### Technical success:

**Figure 2 (A-C):**
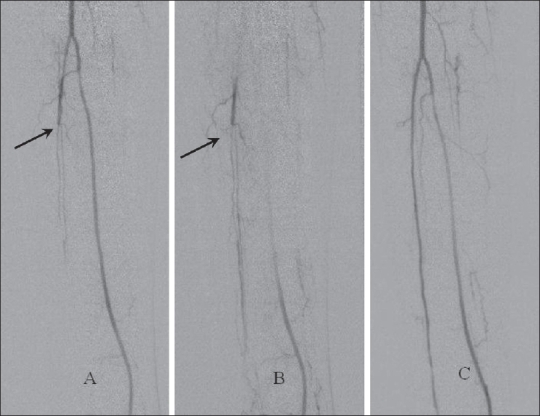
Pre- (A, B) and post-angioplasty (C) angiograms of the leg show an occlusion (arrows) of the middle third of the anterior tibial artery, the occluded segment showing good dilatation following angioplasty. These lesions are not ideal for angioplasty according to TASC guidelines

**Figure 3 (A-D):**
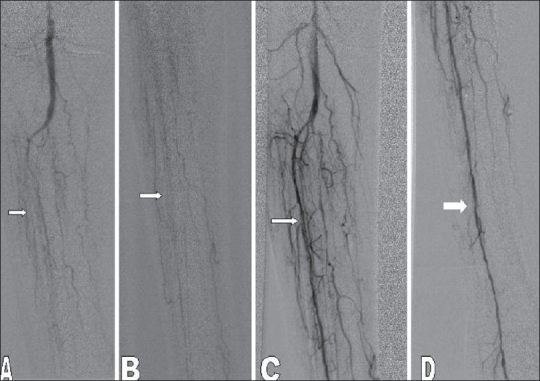
Pre- (A, B) and post (C, D) angioplasty angiograms show a long-segment anterior tibial artery occlusion (arrows) successfully treated with angioplasty (arrows)

The overall technical success rate in our series was 84.7%, which falls within the acceptable range of 78-100% reported in literature.[5] Most of the lesions were not ideal for angioplasty according to current guidelines, since group C and D lesions constituted 87% of the treated lesions [Figure 2]. In a few patients, occluded segments of up to 13 cm length were also dilated [Figure 3]. It appears that a high technical success rate can be achieved even in situations traditionally considered unfavorable for angioplasty.

### Clinical success:

Our results indicate a reasonably good ulcer healing rate (58.62%) and a high limb salvage rate (79.4% at the end of 6 month, 75.8% at the end of 1 year). The ulcer healing rate was lower than the limb salvage rate because 42.8% of our patients presented in a partial/complete gangrenous state. Ulcer healing was not expected in these patients. Angioplasty was performed in these cases for limb salvage, to lower the amputation level and also for stump healing. Of six patients who underwent below-knee amputation despite technically successful angioplasty, five had presented with gangrene and one patient had a grade 3 lesion. All of them had group C or D lesions and no disease-free infrapopliteal vessel (grade 4 ulcer in one patient, grade 3 ulcer in two patients). We infer from these results, that the gangrenous stage is a negative prognostic factor. However the angioplasty helped to heal minor/major amputation stumps. Two forefoot amputation stumps and six below-knee amputation stumps healed well. The ulcer healed in three patients with conservative measures after failed angioplasty. These patients had at least one disease-free infrapopliteal vessel. Probably these disease-free vessels helped in the healing even after a failed angioplasty. One patient died during the follow-up period; the cause of death was not related to the procedure.

Our results are comparable to Saab *et al*.,[7] who reported a clinical success rate of 58-75% in a general population comprising of diabetic and non-diabetic patients. Our results in the diabetic population with extensive group C and D lesions are comparable to the results in a general population. Our results do not support the belief that diabetic patients will not benefit much from infrapopliteal angioplasty.[8,9] Despite a lower patency rate, various studies have shown that infrapopliteal angioplasty in diabetic patients can produce a limb salvage rate comparable to that of bypass surgery, with less morbidity and mortality [Table 5].[3,5,10,12]

**Table 5:** Comparison of bypass surgery and PTA (different sources from literature)[3,5,10,12]

	Bypass surgery (%)	PTA (%)
Survival rate		
1^st^ year	69-88	>90
2^nd^ year	58-68	76
3^rd^ year	44-50	56
Limb salvage		
1^st^ year	81-88	77-89
2^nd^ year	73-88	49-89
3^rd^ year	68-80	72-87
Patency rate		
1^st^ year	81	13-81
2^nd^ year	61	48-78

The cost effectiveness of infrapopliteal angioplasty was evaluated by a multicenter, randomized controlled study (BASIL)[11] conducted by the National Health Service (UK). It concluded that, whenever possible, patients who were expected to live for less than 2 years and had significant comorbidity should be offered angioplasty first. Their conclusion was based on the fact that the outcome after amputation or angioplasty was the same and that, in the short term, surgery was more expensive than angioplasty.

### Complications:

**Figure 4 (A, B):**
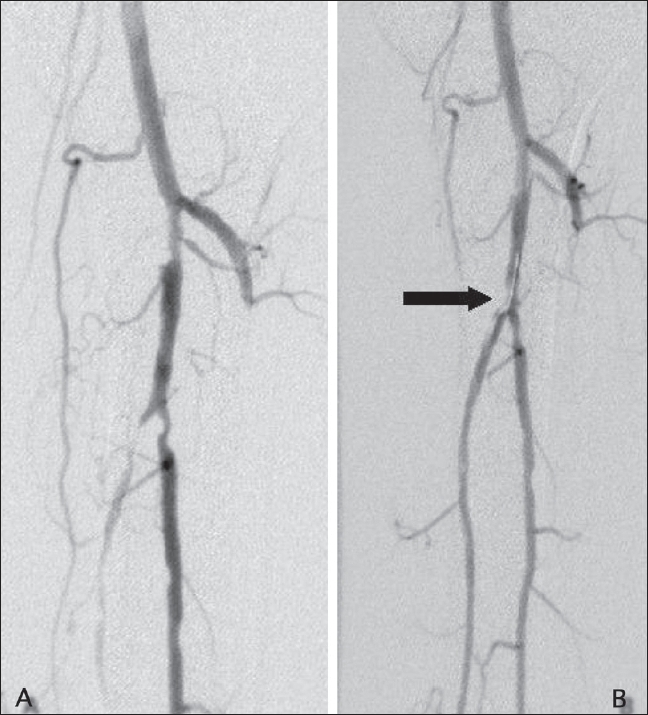
Pre- (A) and post (B) angioplasty angiograms of the distal popliteal artery show a dissection (arrow) extending into the posterior tibial artery origin

**Figure 5:**
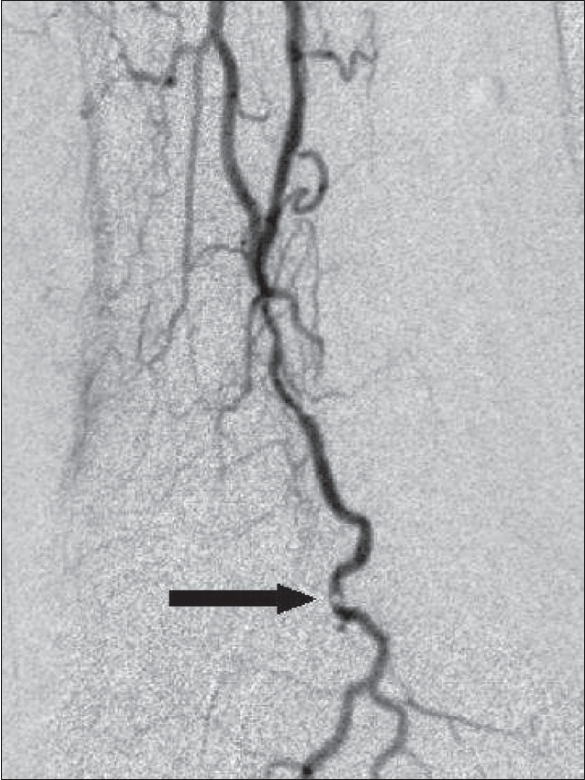
Post-angioplasty angiogram of the leg shows a filling defect (arrow) in the posterior tibial artery, distal to the angioplasty site, suggestive of an embolus

**Figure 6:**
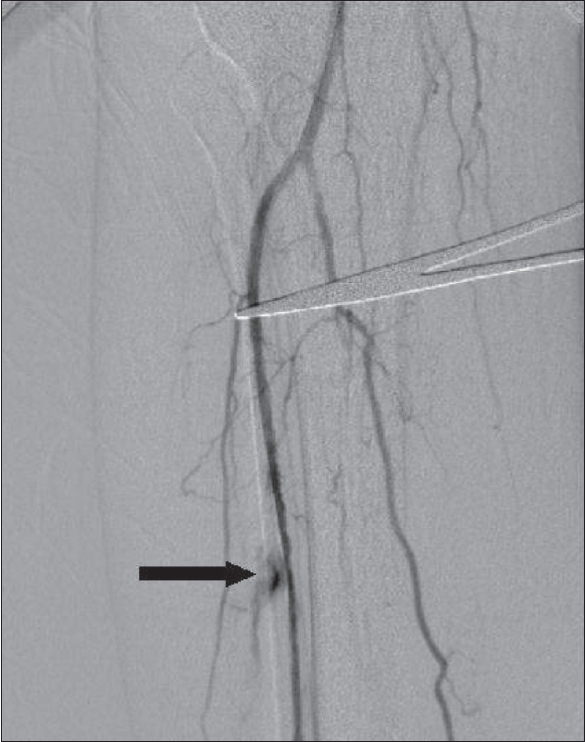
Post-angioplasty angiogram of the leg shows extravasation of contrast (arrow) from the angioplasty site

**Figure 7 (A-C):**
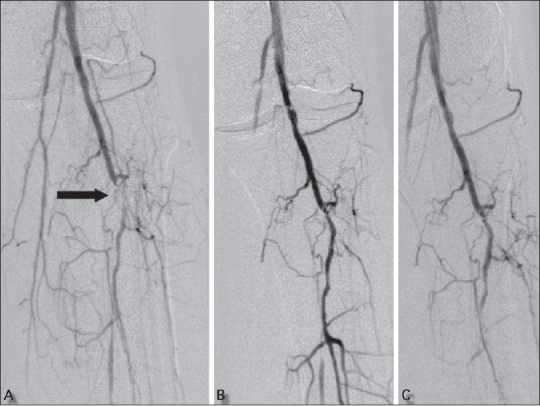
Pre- (A) and post-angioplasty (B, C) images of the distal popliteal artery. The run-off vessel shows vasospasm at the end of the procedure (C)

Small dissections with no luminal compromise, dissections, extravasation, and vasospasm were observed in this study and treated successfully [Figures 4–7]. No secondary stenting was performed. In one patient, post-angioplasty spasm was treated successfully with nitroglycerin. Small non-occluding plaque embolization in one case was left alone, without thrombolysis. We observed one case of transient acute renal failure in a patient who had a high baseline creatinine value (1.7 mg/dl). Immediate reocclusion of the angioplastied vessel occurred in five patients. Groin hematoma has been reported in 5-6% of cases,[12] of whom 20% usually require surgical repair and 20% require transfusions. No puncture site hematoma was encountered in our series

## Conclusion

Poor patency rates do not correlate with higher limb salvage rates [Table 6]. It is sufficient for the angioplasty site to be patent till the ulcer heals. High technical success rates can be achieved even in situations traditionally considered unfavorable for angioplasty. Angioplasty should be employed as the primary management tool in a diabetic foot for the purpose of limb salvage. Infrapopliteal angioplasty can produce a limb salvage rate comparable to that of bypass surgery in diabetic patients with extensive disease.

**Table 6:** Observations in infrapopliteal angioplasties

S No	Criticism of infrapopliteal angioplasties	Our observations
1	Patency rate for small vessel angioplasty is very poor	Short-term patency of infrapopliteal arteries is good enough for ulcer healing Long-term patency is not relevant clinically since limb-threatening ischemia may not recur
2	Diabetics have longer lesion which are not ideal for angioplasties according to TASC guide lines	It is worth attempting infrapopliteal angioplasty for limb salvage purposes Technical success rates are encouraging even in longer lesions due to newer, better hardware
3	Surgical bypass produces better results	Infrapopliteal angioplasty not only produces comparable clinical out come but does so with lesser morbidity and mortality
